# A deep learning dataset for sample preparation artefacts detection in multispectral high-content microscopy

**DOI:** 10.1038/s41597-024-03064-y

**Published:** 2024-02-23

**Authors:** Vaibhav Sharma, Artur Yakimovich

**Affiliations:** 1 Center for Advanced Systems Understanding (CASUS), Görlitz, Germany; 2 Helmholtz-Zentrum Dresden-Rossendorf e. V. (HZDR), Dresden, Germany; 3 Artificial Intelligence for Life Sciences CIC, Dorset, UK; 4 Institute of Computer Science, University of Wroclaw, Wroclaw, Poland

**Keywords:** High-throughput screening, Signal processing

## Abstract

High-content image-based screening is widely used in Drug Discovery and Systems Biology. However, sample preparation artefacts may significantly deteriorate the quality of image-based screening assays. While detection and circumvention of such artefacts could be addressed using modern-day machine learning and deep learning algorithms, this is widely impeded by the lack of suitable datasets. To address this, here we present a purpose-created open dataset of high-content microscopy sample preparation artefact. It consists of high-content microscopy of laboratory dust titrated on fixed cell culture specimens imaged with fluorescence filters covering the complete spectral range. To ensure this dataset is suitable for supervised machine learning tasks like image classification or segmentation we propose rule-based annotation strategies on categorical and pixel levels. We demonstrate the applicability of our dataset for deep learning by training a convolutional-neural-network-based classifier.

## Background & Summary

Image-based phenotypic screening is a widely used approach for early hit identification in screening-based drug discovery employing automated high-content microscopy^1–3^. Furthermore, combined with scalable cell culture assays, image-based high-content screening provides a rich source of big data in systems biology allowing to deduce molecular mechanisms at a genome-wide scope^4–7^. Such versatility is largely facilitated through the advances in fluorescence microscopy^8^, as well as fluorescent dyes and labels^9,10^ capable of visualising virtually any molecule inside of the cell. Combined with the latest achievements in biomedical image analysis, machine learning (ML) and deep learning (DL), high-content imaging promises to employ its big-scale capacity to render a platform for end-to-end biomedical discovery.

However, as sample preparation and image acquisition become more automated the absence of the “invisible hand” of the microscopist becomes more obvious^11^. Thanks to the abundance of manually curated datasets obtained by highly-trained microscopists, many existing fluorescence image analysis algorithms^12^ simply don’t take into account the presence of sample preparation artefacts (SPA). SPA can occur during cell culture, fixation, staining, mounting or other sample preparation steps due to various mechanical and/or chemical interactions. Also, SPA may arise due to the presence of unwanted dust, precipitates or contaminants in the experimental environment. SPAs are especially common in automated liquid handling, or sample preparation, which, in turn, is widely used in high-content or screening microscopy^3,11^.

The presence of such artefacts in the resulting micrographs may inevitably introduce errors in quantification or invalidate scientific conclusions obtained using such images. While using SPA-containing images for analysis may be easy to avoid by a well-experienced microscopist, automated systems or ML/DL-powered systems are rarely trained to recognize SPA. With the advent of Computer Vision and ML/DL for automated microscopy quantification, errors resulting from the presence of SPA in large datasets may, in turn, affect the downstream steps of biomedical discovery like a pathway or drug discovery, vaccine development etc. It must be noted, that artefacts resulting from optical aberrations may be removed by image reconstruction algorithms using a forward model of a microscope, e.g. models of point spread function. However, since SPA are physical objects inside the images, it is not possible to use the forward model of a microscope to remove them^13^. DL-based Computer Vision methods^14,15^ could be used to detect artefacts inside microscopic images, however, there is no publicly available dataset which resembles experimentally-relevant SPA which hinders the applicability of DL methods as a solution to this problem.

To address this, here we present a purposefully collected open dataset (Creative Commons Attribution 4.0 International) of HeLa cells cultured in a multi-titre plate and imaged using a high-content fluorescence microscope. To simulate the presence of SPA we have collected and titred laboratory dust. To ensure that we cover all of the aspects of SPA autofluorescence we acquired images with filter assemblies suited for multiple parts of the light spectrum (multispectral images) ranging from ultra-violet to the far-red parts of the spectrum. Finally, to ensure that this dataset is suitable for ML/DL-powered image analysis we propose an approach for weak labelling (rule-based labelling) of the artefacts and train an artefact detection model based on a multi-layered convolutional neural network (CNN). We argue that the dataset we provide here will be of great value to the biomedical image analysis community and will serve to develop a new generation of more robust ML/DL models.

## Methods

### Cell culture and sample preparation

**Fig. 1:**
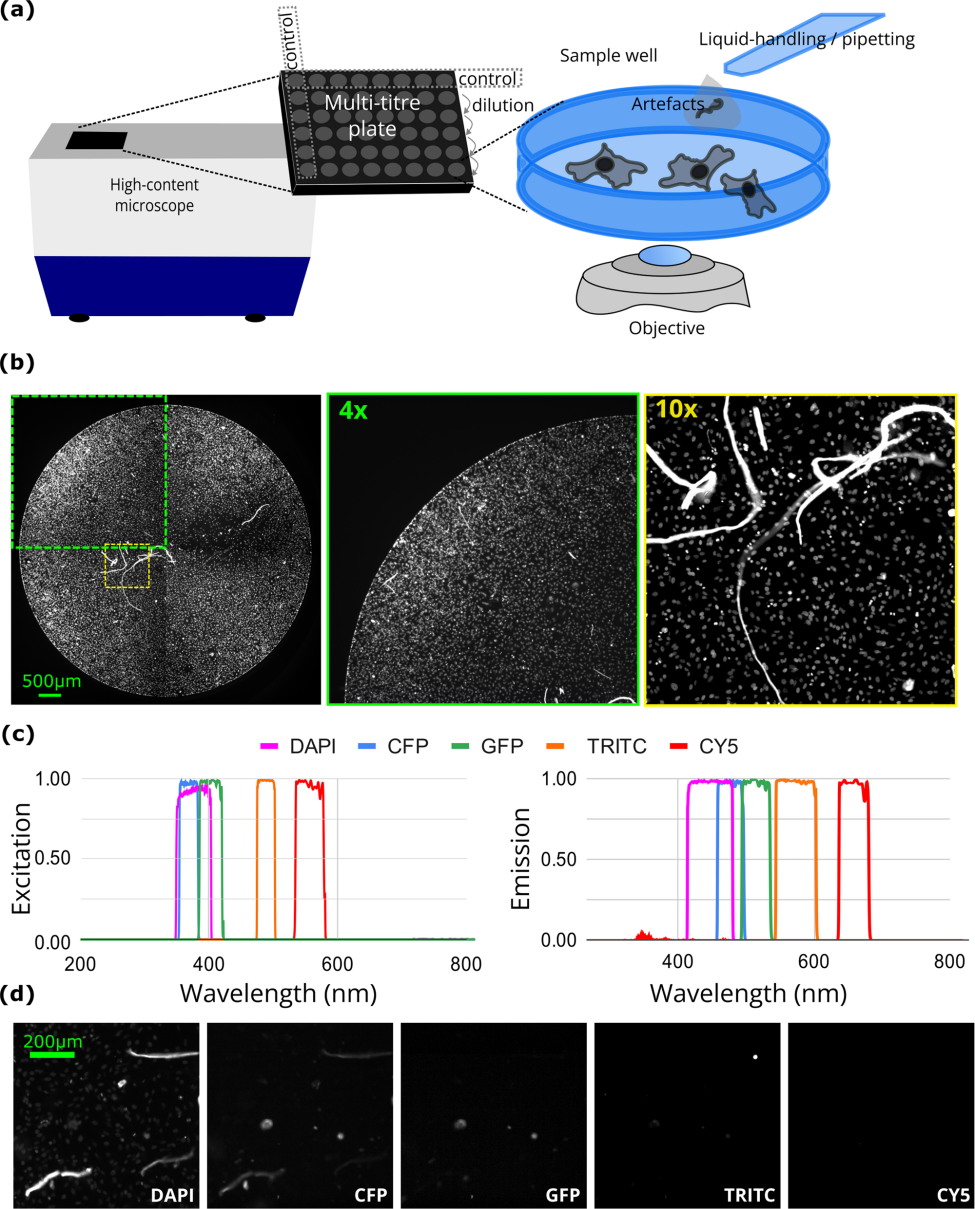
Multispectral high-content imaging dataset of cultured HeLa cells with sample preparation artefacts. (**a**) Image acquisition diagram depicting a 96-well imaging plate and indicating the source of the sample preparation artefacts. (**b**) An example of a reconstructed full well image from the DAPI channel, as well as 4x (30 ms exposure) and 10x (9 ms exposure) magnification image respectively. Scale bar 500 µm. (**c**) A diagram showing the excitation and emission wavelengths of the filter cubes used. (**d**) Sample images from different spectral channels described in panel c. Scale bar 200 µm.

To mimic a high-content image-based screening experimental setup we have used a black 96-well (rows A to H and columns 1 to 12) polystyrene imaging plate (CLS3603-48EA, Corning, Sigma) containing cultured HeLa ATCC cells (Fig. 1b). Cells were seeded a day prior to the experiment in 200 µL volume (per well) containing 250000 cells per mL in Dulbecco’s Modified Eagle’s Medium (Sigma) containing 4500 mg/L glucose (Sigma), L-glutamine (Sigma), sodium bicarbonate (Sigma), sodium pyruvate (Sigma), 10% foetal calf serum (Sigma) and non-essential amino acids (Sigma). To obtain varying cell density the cell suspension was diluted during seeding at a 1 to 2 ratio from columns 2 to 12. Column 1 was reserved as no-cells control. Cells were incubated overnight at 37 °C with humidity control and 5% CO_2_. On the next day, cells were fixed with a 4% paraformaldehyde (Sigma) solution in phosphate buffer saline (PBS, Sigma). Next, cell nuclei were stained with a 40 µg/mL solution of Hoechst 33342 dye (Sigma). Row A was kept unstained as control (Fig. 1a, Table 1).

**Table 1:** 96-well plate treatment and controls overview.

	01	02	…	11	12
A	No cells; No Hoechst	No Hoechst	No Hoechst	No Hoechst	No Hoechst
B	No cells	High artefact count (1:1 dilution)	High artefact count (1:1 dilution)	High artefact count (1:1 dilution)	High artefact count (1:1 dilution)
…	…	…	…	…	…
G	No cells	Low artefact count (1:32 dilution)	Low artefact count (1:32 dilution)	Low artefact count (1:32 dilution)	Low artefact count (1:32 dilution)
H	No cells; No artefacts	No artefacts	No artefacts	No artefacts	No artefacts

Here Hoechst refers to Hoechst 33342 nuclear dye (see Methods). At 1:1 dilution artefacts cover approximately 3% of the well.

Upon preparation of the *bona fide* artefact-free experimental plate, we collected samples of dust across the approximately 100 m^2^ laboratory and prepared a suspension of these dust samples in PBS. This suspension was then added to rows A to G in a serial dilution manner, with row H as the control (Fig. 1a, Table 1).

### High-content multispectral microscopy

High-content multispectral microscopy images were obtained with an automated ImageXpress XL epi-fluorescence microscope (IXM XL, Molecular Devices) using either a 10x Nikon Plan Fluor objective with a 0.3 numerical aperture (NA) or 4x Nikon S Fluor objective (for the schematic depiction of the imaging setup see Fig. 1a,b) with a 0.2 NA. IXM XL was equipped with multiple wavelength excitation/emission filters (Semrock). Each well was imaged with fields of view (sites). The full well was imaged in 4 sites at 4x magnification. To enable the acquisition, IXM XL was equipped with an automatic motorised stage, laser-based autofocusing, 16-bit pco.edge sCMOS camera, fluorescence filter cubes and a diode light source enabling imaging at 5 different wavelengths. Image size was 4.66 megapixels covering the field of view 3.5 × 3.5 mm at 4x and 1.4 × 1.4 mm at 10x. IXM XL was equipped with undimmable LED light source and a digital shutter allowing to control the illumination intensity exclusively via exposure time.

The images in this multispectral dataset contain information from five wavelengths obtained using filter cube assemblies (Semrock) adopted for the following fluorophores: cyan fluorescence protein (CFP, CFP-2432C-NTE-ZERO), cyanine5 (CY5, Cy5-4040-NTE-ZERO), 4,6-diamidino-2-phenylindole (DAPI, DAPI-5060C-NTE-ZERO), tetramethyl-rhodamine-isothiocyanate (TRITC, TRITC-A-NTE-ZERO) and green fluorescent protein (GFP, GFP-3035D-NTE-ZERO). Each filter cube is characterised by its own emission and excitation transmission which dictates the values for its corresponding filtered wavelength ranges. The emission and excitation ranges for the abovementioned spectral filters are depicted in Fig. 1c, with corresponding image examples shown in Fig. 1d.

### Data preprocessing and annotation

**Fig. 2:**
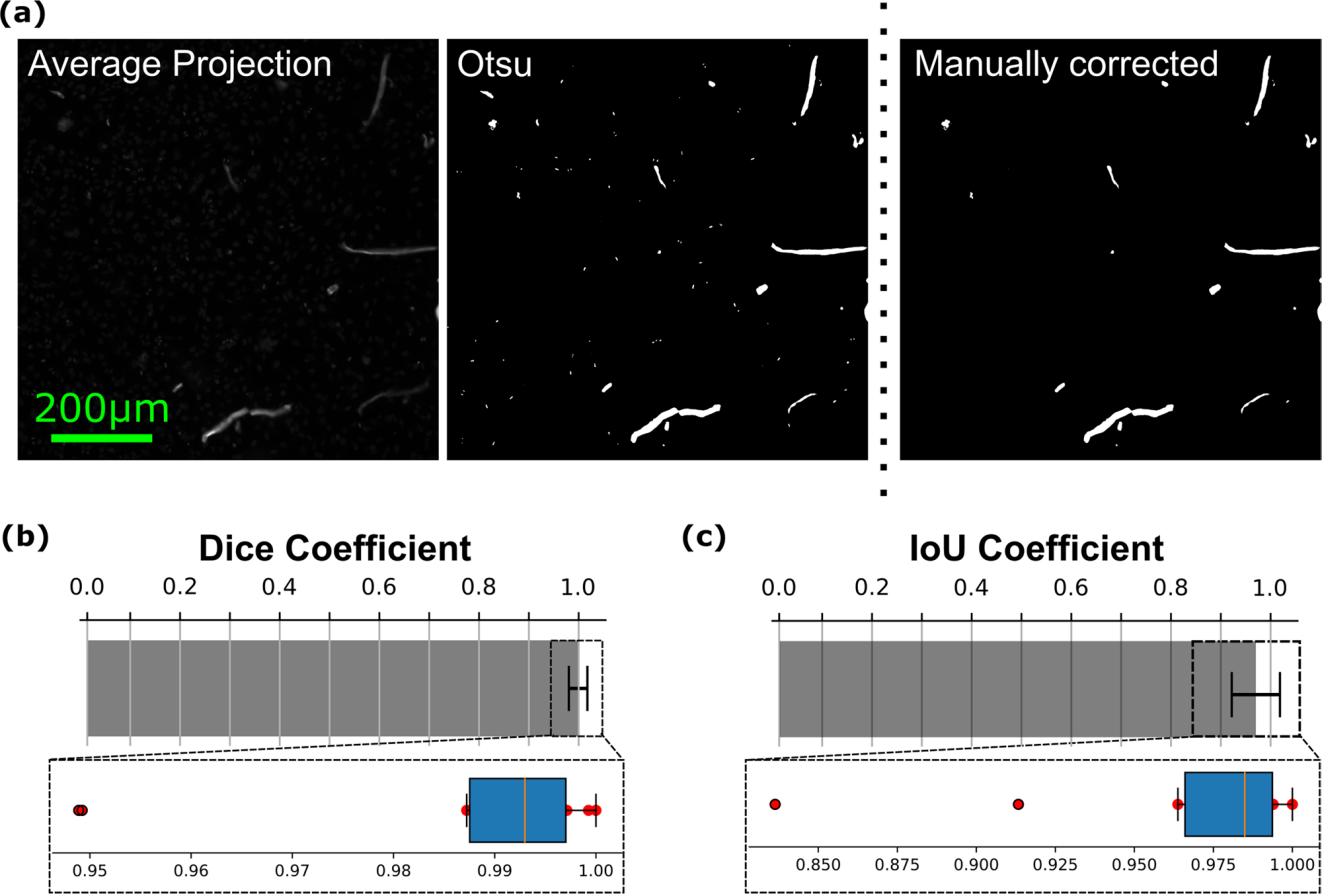
Automated sample preparation artefact detection through multi-exposure image averaging. (**a**) Cyan Fluorescent Protein fluorescence filter averaged image example, Otsu thresholding and manual correction of sample preparation artefact mask respectively. (**b**) Dice coefficient comparison between manual ground truth and Otsu segmented masks. Zoomed inset shows the region designated by the dashed line as a box plot. (**c**) Intersection over union (IoU) coefficient comparison between manual ground truth and Otsu segmented masks. Zoomed inset shows the region designated by the dashed line as a box plot. Error bars indicate standard deviation across images.

The HeLa cells dataset contains images of size 2160 by 2160 pixels. These relatively large images were split into patches of size 256 by 256 pixels. The resulting smaller images are compatible with deep neural network training purposes. There are two levels of annotations that can be obtained from the abovementioned smaller images dataset: categorical (“Artefact”, “Nuclei”) and pixel-level (masks). To obtain pixel-level annotation, we took the average projection of images captured using multiple exposure times (which are denoted by “_w1”, “_w2” and so on, up to “_w6”) from the CFP channel. Performing Otsu thresholding^16^ on these average projection images generated masked images containing only artefacts and some mitotic cells observable by the presence of characteristic chromatin patterns (see Fig. 2). Specifically, due to the high density of chromatin, the DNA in these cells appears as thin bright spots. These mitotic cells were subsequently removed manually by a microscopy specialist.

### Convolutional neural network design and hyperparameters optimisation

**Fig. 3:**
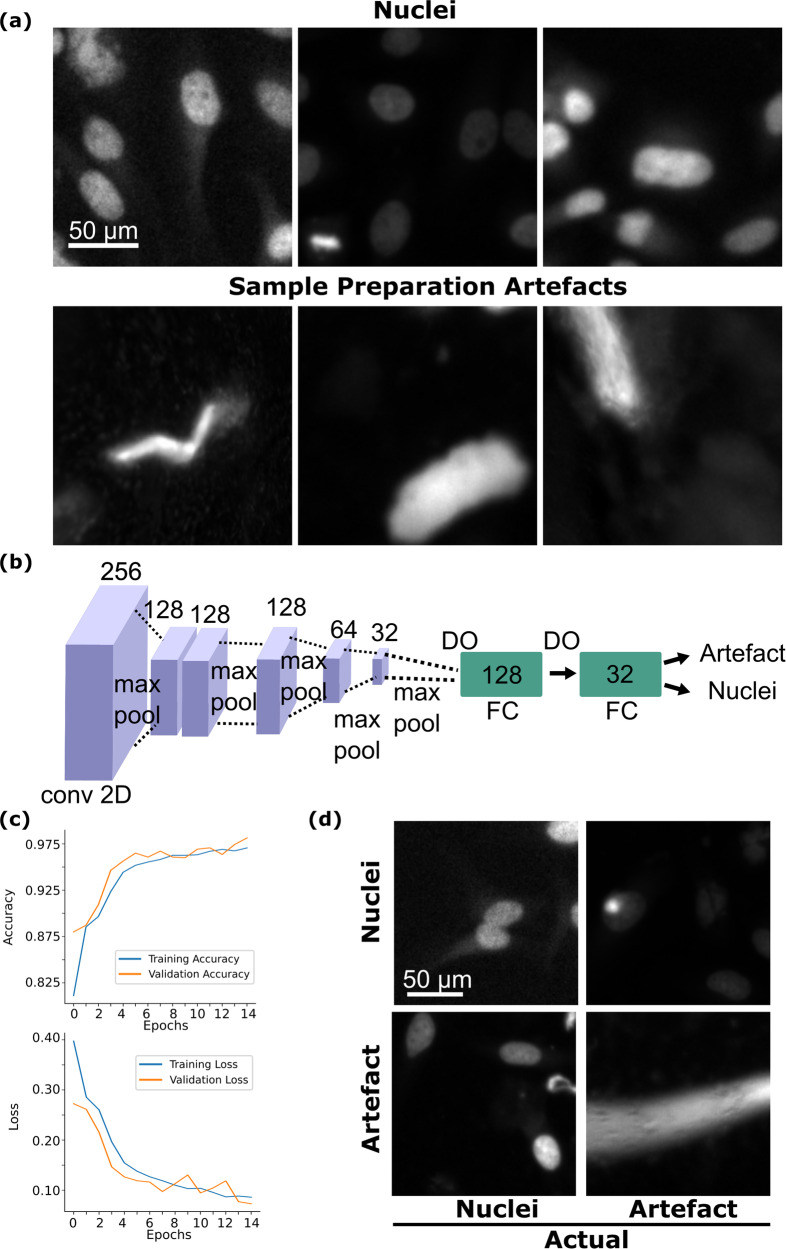
Artefact classifier model description and results. (**a**) Nuclei and sample preparation artefact class examples (**b**) Artefact classifier model architecture. Here, numbers indicate the dimensions of the layers of the convolutional neural network. Conv2D stands for two-dimensional convolutional layer. FC stands for fully-connected layer. DO stands for drop-out regularisation layer. (**c**) Training and validation losses and accuracies for the artefact classifier model. (**d**) Pictorial depiction of the confusion matrix for the artefact classifier model.

The convolutional neural network (CNN) used for artefact classification had 6 (six) 2D convolutional layers (of arranged as 252 × 252 × 256, max pooling, 124 × 124 × 128, 122 × 122 × 128, max pooling, 59 × 59 × 128, max pooling, 27 × 27 × 64, max pooling and 11 × 11 × 32, max pooling, dropout, fully-connected 128, dropout, fully-connected 32, fully-connected classifier) followed by a densely-connected network having 3 layers of sizes 128, 32, 2 (see Fig. 3). The final layer is used to give out a binary classification result with classes: ‘Artefact’ and ‘Nuclei’. Hyperparameters like the number of convolutional layers, learning rate, dropout probability etc. were tuned iteratively to achieve around 98% validation accuracy on the unseen validation data holdout. Namely, training runs with specific configurations were performed and validation performance was observed.

### Convolutional neural network training

To train the CNN we used a single 4 GB NVIDIA GeForce GTX 1050 graphics card, 8 GB of RAM and 4 CPU cores on an Intel i5 5th Generation processor. The model was trained for 15 epochs.

## Data Records

The high-content microscopy SPA dataset was obtained to facilitate the next generation of bioimage analysis tools robust to the influence of SPA. The dataset is available under Creative Commons Attribution 4.0 International licence and can be obtained from^17^. It consists of images obtained with 4x and 10x objectives using fluorescence cube assemblies for DAPI, CFP, GFP, TRITC and Cy5 channels. Each subset contains 384 unique 2160 × 2160 pixels-wide sites (see Fig. 1b–d). For hardware reasons, images with the CFP filter cube were obtained separately from images with DAPI, GFP, TRITC and Cy5 filter cubes. Furthermore, CFP images (and in some cases DAPI images) were obtained with varying exposure times corresponding to “_w1”, “_w2” and so on filename suffixes. This concerns folders C and D, below. Images were obtained using ImageXpress Micro XL high-content microscope (Molecular Devices, see Methods section). Images are organised into the following folders:

A. 4x-cfp
B. 4x-dapi-gfp-tritc-cy5
C. 10x-6cfp
D. 10x-6dapi
E. 10x-cfp
F. dapi-gfp-tritc-cy5
G. filters_spectra

Here, folders A and B correspond to 4x magnification and contain images obtained with the CFP (folder A) and the other filter cubes respectively (folder B). Each folder contains a “TimePoint_1” subfolder containing the raw images. In the case of 4x images, each field of view (“site” designed with “_s1”, “_s2” etc. suffixes) corresponds to a nearly perfect quarter of a 96-well plate well. In addition to the raw images in the “TimePoint_1”, a subfolder “Stitched” contains images of the entire wells. In the case of folder B containing all other fluorescence channels, “_w1”, “_w2”, “_w3”, and “_w4” correspond to a single optimal exposure time of DAPI, GFP, TRITC and Cy5 filters respectively.

Similarly, folders C - F correspond to 10x magnification and contain images of multiple exposures of CFP and DAPI (folders C and D) and single exposures of CFP and other channels (folders E and F). In the case of CFP and DAPI multiple exposure folders, varying exposure times correspond to “_w1”, “_w2” etc. Six different exposure times allow to evaluate SPAs under varying illumination. Finally, folder G contains metadata on filter cubes used in the dataset, including the emission and excitation filter spectra for each filter cube. To ensure both artefact-containing and artefact-free images are present in the dataset, upon *bona fide* clean plate preparation a serial 1:2 dilution of laboratory dust samples suspended in buffer was added to the wells. Laboratory dust samples were aimed to represent one of the major sources of SPA, hence allowing the presence of the SPA to be quasi-concentration-dependent. As a result, row B of the plate contains the highest concentration of SPA, while row G - the lowest, while row H was kept as an artefact-free control. A full overview of the plate arrangement and controls can be found in Table 1.

## Technical Validation

### Data annotation and preprocessing

To ensure that our SPA dataset is suitable for application in supervised learning we proposed methods to obtain annotations at two levels. Firstly, since some wells did not contain any SPA altogether by the experimental design, categorical annotations (i.e. Artefact/Nuclei) may be readily obtained from the file name of the image (see Table 1). Secondly, to obtain pixel mask level annotations for SPA, the multispectral and multi-exposure nature of the dataset was utilised. Specifically, as the Hoechst fluorescent nuclear dye tends to emit light closer to the UV part of the spectrum, sampling from the remaining blue, green, red and far-red parts of the spectrum are more likely to contain information from the artefacts autofluorescence (e.g. fibres and microplastics autofluorescence).

Upon examination of the information present in the data, we have realised that the vast majority of the autofluorescence information was present in the DAPI and CFP channels. At the same time since the DAPI channel was also used for our target nuclear staining, we have opted to employ mostly the information present in the CFP channel. To harness this, we have obtained an average projection from multiple images with varying exposure times obtained in CFP and applied the Otsu thresholding algorithm^16^ to obtain a binary mask of the artefacts (Fig. 2a, see Methods). Next, to assess the correctness of the masks obtained in such a manner, we have compared them to manually annotated masks of the artefacts. We then used the Dice coefficient and IoU scores for Otsu thresholded images to compare the average-projection-based annotations to the manual ground truth images with respect to the 2160 × 2160 pixel images (Fig. 2b,c). We concluded that this approach provides a good way to obtain weak mask-level annotation for the SPA dataset.

Finally, to ensure that the dataset can be used for machine learning we devised an image preprocessing approach. In our approach, each individual micrograph measures 2160 × 2160 pixels and can be used to generate multiple individual patches downstream (e.g. 256 × 256 pixels). This step not only allows to optimise performance while retaining high resolution but also allows for a significant boost in the size of the ML/DL dataset in a strategy known as data augmentation^15,18^.

### Artefact classifier

To show that a CNN-based classifier can be readily trained using our dataset, we have designed a CNN-based image classifier employing categorical annotations. Specifically, we used the file names of the images to ensure they come from the wells containing Nuclei to generate the ground truth data for the proposed classifier model (Fig. 3a). For the sake of demonstration, this model was designed to classify input images into two classes: “Artefacts” or “Nuclei”. The architecture of our proposed artefact classifier consists of a four-layered CNN followed by a dense classification layer connected by a max-pooling layer. The final layer contains two output neurons with softmax activation to classify the input images into our two target classes (Fig. 3b, see Methods).

The model was trained using patches (256 × 256) generated from the HeLa cells multispectral dataset in which each image is 2160 × 2160 pixels in size. To train the illustration model, we have used 16000 patches, split into train validation and test holdouts at 0.79:0.09:0.12 ratio. Sample preparation artefacts have been deliberately added upon preparation of the plate (see Table 1). As mentioned above, we used this to obtain categorical annotations allowing us to split the images into two target classes: “Nuclei” and “Artefact”. Figure 3a shows samples of the ground truth images used for the classifier training.

Upon conclusion of the training our model achieved a training accuracy of 97.06% and a validation accuracy of 98.14% (Fig. 3c), test accuracy was 91.25%. Despite minor overfitting, the high validation and test accuracy suggests that the model is highly effective at filtering out image artefacts. While the performance on the training and validation holdout sets was promising, to address the potential effects of the class imbalance we also examined precision, recall and the F1 score on the test holdout (Table 2). Given the high values of the precision and recall, to further validate our model we have examined the confusion matrix (Table 3), where the positive class was “Nuclei” and the negative class - “Artefacts”. To obtain a visual impression of the confusion matrix obtained from the classifier we have also depicted representative patches in Fig. 3d.

**Table 2:** Test Performance of the Sample Preparation Artefact Classifier.

	Precision	Recall	F1- Score
Artefact	0.977	0.845	0.906
Nuclei	0.863	0.980	0.918

The “Positive” class is “Nuclei” and the “Negative” is “Artefact”.

**Table 3:** Confusion Matrix of the Artefact Classifier model.

	Positive	Negative
Positive (true)	845	155
Negative (true)	20	980

Here, “Positive” is “Nuclei” and “Negative” is “Artefact”.

### Validation conclusions

Presence of SPA in high-content microscopy datasets may significantly influence the performance of bioimage analysis algorithms. In this work, we acquired and characterised an open dataset aimed to facilitate data-driven algorithms robust to SPA in large microscopy datasets. To ensure that this dataset can be used for supervised learning we have proposed annotation approaches at two levels: whole-image-based categorical annotations for classification task and pixel-level mask annotations for image segmentation task. While categorical annotations were possible by experimental design, pixel-level annotations are possible through the utilisation of the multispectral nature of the dataset. We argue that the latter broadens the applicability of our dataset beyond cell nuclei imaging. Furthermore, we have validated the applicability of our SPA dataset for ML/DL by training an image classifier. This classifier is immediately available to the community via the code repository (https://github.com/casus/deepdedust). Additionally, the open-source code accompanying this paper can be readily used as a primer by other researchers to develop their own respective applications for our SPA dataset.

Despite the seeming simplicity, the proposed classifier could potentially be used for quality control during microscopy. This direct artefact-free dataset generation from the microscope can be achieved in two steps. First, a single large image (generally above 1024 × 1024 pixels) taken by a microscope can be fragmented into smaller patches of zoomed images of desirable dimensions using a zoomed image generator. This down-sampling step is necessary because most DL-based models have exponential time complexity with respect to the input image size. Second, the resulting patches (zoomed images) can then be fed to the trained artefact classifier which would filter out most of the zoomed images containing artefacts. The filtering accuracy will depend on the accuracy of the classifier model.

## Data Availability

All the code developed for this work is available under an open-source MIT license. It can be found at https://github.com/casus/deepdedust.
